# Case Report: Usefulness of Biomarkers for Alzheimer's Disease in Two Cases With Very-Late-Onset Schizophrenia-Like Psychosis

**DOI:** 10.3389/fpsyt.2021.742659

**Published:** 2021-09-14

**Authors:** Yuto Satake, Hideki Kanemoto, Kenji Yoshiyama, Ryoko Nakahama, Keiko Matsunaga, Eku Shimosegawa, Takashi Morihara, Mamoru Hashimoto, Manabu Ikeda

**Affiliations:** ^1^Department of Psychiatry, Osaka University Graduate School of Medicine, Osaka, Japan; ^2^Department of Child and Adolescent Psychiatry, Osaka City General Hospital, Osaka, Japan; ^3^Department of Molecular Imaging in Medicine, Osaka University Graduate School of Medicine, Osaka, Japan; ^4^Department of Neuropsychiatry, Kindai University Faculty of Medicine, Osaka, Japan

**Keywords:** prodromal Alzheimer's disease, amyloid PET, cerebrospinal fluid, very-late-onset schizophrenia-like psychosis, case report

## Abstract

The association between primary psychotic disorders emerging in later life and neurodegenerative diseases, including Alzheimer's disease (AD), is controversial. We present two female non-demented cases of psychosis with onset above the age of 60 years. Cases 1 and 2 were aged was 68 and 81 years, respectively. They suffered from persecutory delusions and scored 28 on the Mini-Mental State Examination (MMSE) at the first examination. Although detailed neuropsychological tests detected amnesia, they had preserved daily life function. Brain magnetic resonance imaging, N-isopropyl-p-[123I] iodoamphetamine (123I-IMP) single-photon emission computed tomography, and cardiac [123I]-metaiodobenzylguanidine (123I-MIBG) scintigraphy showed no specific abnormalities in either case. We diagnosed them with very-late-onset schizophrenia-like psychosis (VLOSLP) because there was no evidence that their psychoses were derived from organic diseases or affective disorders. Upon close inspection, the AD biomarkers, cerebrospinal fluid (CSF) testing and Florbetapir F 18 positron emission tomography (PET), were positive in Case 1 and negative in Case 2. Case 1 scored 25 1 year later and 23 2 years later on the MMSE and was finally diagnosed as AD dementia. These two cases suggest that some clinically diagnosed VLOSLPs may be a prodromal AD. Although VLOSLP is a disease entity supposed to be a primary psychotic disorder, some are probably secondary psychosis with insidious neurodegeneration. Advanced biomarkers such as amyloid PET and CSF may contribute to the detection of secondary psychosis from clinically diagnosed VLOSLP.

## Introduction

Since the 1940's, many researchers have been interested in late-onset primary psychosis (1–4) due to several phenomenological or psychosocial characteristics that differ from those of average-onset schizophrenia spectrum disorders. Late-onset primary psychosis has a clear preponderance in women living alone (5), and patients often present with persecutory delusions and few negative symptoms (6). Therefore, late-onset primary psychosis may have a different etiology from that of average-onset schizophrenia spectrum disorders. In 2000, the comprehensive disease entity “very-late-onset schizophrenia-like psychosis (VLOSLP)” was proposed (7) and has since been investigated thoroughly. Several neuropathological studies have shown that Lewy body disease (LBD), argyrophilic grain disease (AGD), and primary age-related tauopathy (PART) are associated with late-onset schizophrenia and VLOSLP (8, 9). In addition, several longitudinal reports have shown that some patients with VLOSLP develop dementia (10–12). Therefore, neurodegeneration may be a crucial factor for developing VLOSLP.

At the onset of psychosis, it is difficult to predict the pathology because of the lack of symptoms associated with organic diseases, which is a requirement for diagnosis. However, there have been remarkable advances in biomarkers to assess pathology in live patients. Cerebrospinal fluid (CSF) biomarkers and amyloid positron emission tomography (PET) are available to diagnose Alzheimer's disease (AD), even at the prodromal and preclinical stages (13). Further, 2β-carbometoxy-3β-(4-iodophenyl)-N-(3-fluoropropyl) nortropane (FP-CIT) single-photon emission computed tomography (SPECT), assessing the loss of presynaptic dopamine transporters, and cardiac metaiodobenzylguanidine (MIBG) scintigraphy, detecting the dysfunction of myocardial postganglionic noradrenergic neurons, are available as LBD biomarkers. Despite the lack of evidence in VLOSLP, such biomarkers are also expected to be reliable at the prodromal stage (14, 15). Herein, we report two cases of VLOSLP examined using AD and LBD biomarkers. One patient was positive for AD biomarkers and negative for LBD biomarkers, whereas the other was negative for both AD and LBD biomarkers.

## Case Description

Both our cases had persistent persecutory delusions and slight amnesia. As their cognitive deficits and symptom presentations were so similar, we selected them for comparison.

[Case 1] The female patient was 68 years old at her first visit. She was a left-handed high school graduate. She lived with her husband and did not have any children. She experienced only an extra-uterine pregnancy as past medical history and had no psychiatric or neurological family history. Four months before her visit, she got a persecutory delusion wherein her neighbors entered her house during her absence and followed her everywhere. She harbored the idea of finding something in an unexpected place and thinking that someone had moved it during her absence. She also began to become sensitive to lights in her daily life and said that someone cast flashes at her. Because her delusional ideas disrupted her daily life, she received clotiazepam for her anxiety from her primary doctor before her first referral to our hospital, where she was hospitalized under close examination for diagnosis.

We did not find any apparent memory deficits, cognitive fluctuation, altered nighttime sleep behaviors, depressive mood, manic state, or hallucinations during hospitalization. She presented with extreme anxiety; thus, she spent most of the time in the ward's day room. She often felt that something scary would happen, which was considered a delusional mood. She also seemed to have the delusion of observation. However, her psychotic symptoms were not severe.

**Figure 1:**
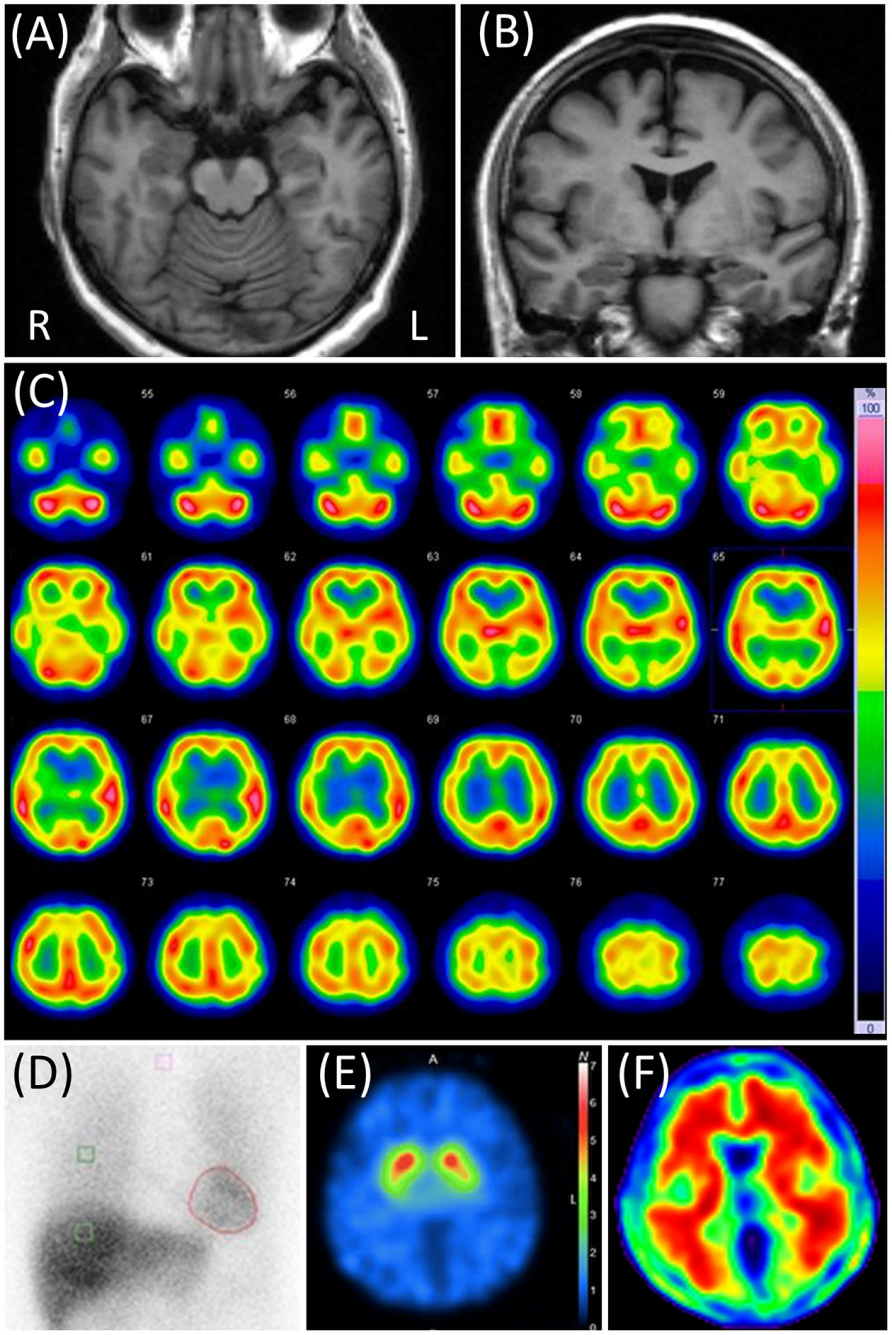
Case 1 images. **(A,B)** Brain magnetic resonance T1-weighted images. None of them showed any abnormalities. **(C)** N-isopropyl-p-[123I] iodoamphetamine (123I-IMP) single-photon emission computed tomography (SPECT) not showing any particular abnormalities. **(D)** Cardiac [123I]-metaiodobenzylguanidine scintigraphy. Heart-to-mediastinum ratios were 3.33 and 2.28 in early and delayed phases, respectively. The washout ratio was 48.8%. **(E)** 2β-carbometoxy-3β-(4-iodophenyl)-N-(3-fluoropropyl) nortropane (FP-CIT) SPECT. The specific binding ratios were 6.53 and 6.04 in the right and left striatum, respectively. The asymmetry index was 7.9%. **(F)** Florbetapir 18 positron emission tomography showing positive results.

Neurological examination, blood test, and electroencephalography findings were normal. The CSF cell count and the protein concentration were 0/μL and 35 mg/dL, respectively. The Mini-Mental State Examination (MMSE) score was 28 (2 points lost in the task of serial 7's), and Logical Memory (LM) in the Wechsler Memory Scale-revised (WMS-R) was 11 (immediate recall)/1 (delayed recall). The four subtests (Digit Symbol Substitution, Block Design, Digit Span, and Information) of the Wechsler Adult Intelligence Test III (WAIS-III) and the Alzheimer's Disease Assessment Scale-cognitive component-Japanese version (ADAS-J cog) did not reveal any cognitive impairments. The neuropsychiatric Inventory-12 (NPI-12) revealed anxiety, apathy, difficulty falling asleep, and appetite loss, apart from delusions. Brain magnetic resonance imaging (MRI) showed almost no visually-rated cerebral atrophy, no cerebrovascular lesions, and no white matter T2 hyperintensities. 123I-IMP SPECT and cardiac 123I-MIBG scintigraphy showed no specific abnormalities (Figure 1).

Based on the above results, we diagnosed the patient with VLOSLP. We judged that the mild amnesia found in LM was attributed to her psychosis and anxiety at the time. As her symptoms were not severe during hospitalization, she was discharged without new prescriptions. Because her psychosis recurred after discharge, her husband began to stay with her as much as possible to ease her anxiety.

In the CSF testing, phosphorylated tau at epitope 181 (p-tau) and total tau (t-tau) were 86 and 697 pg/mL, respectively, as measured by SRL, Inc. using a previously reported method (16, 17). Those results were reported within 4 weeks after the discharge. Apolipoprotein E (APOE) genotyping showed ε4/ε3. All these results were suggestive of AD pathology (18). one year later, 123I-FP-CIT SPECT was performed. The specific binding ratio was 6.53 and 6.04 in the right and left striatum, respectively, suggesting a negative result. Florbetapir F 18 PET was positive (Figure 1). Based on this evidence, she received a diagnosis of AD pathology.

During follow-up, her MMSE score dropped to 25 (three points lost in the task of delayed recall and two points lost in the serial 7's) 1 year later and to 23 (three points lost in the delayed recall, two points lost in the serial 7's, and two points lost in the orientation of time) 2 years later. She gradually developed apparent recent memory impairments in her daily life and finally was incapable of shopping and cooking by herself 2 years after her first visit. We finally diagnosed her with AD dementia and started rivastigmine as treatment for her cognitive deterioration. On the other hand, her psychosis and anxiety had diminished compared to her first visit, as did her delusional mood and delusion of observation, and she just repeatedly reported that there were unfamiliar things at home. However, 2 years after her first visit, she began to present a mild delusion of theft.

[Case 2] A female patient visited our hospital at the age of 81 years. She was a right-handed high school graduate. She lived with her husband with severe Parkinson's disease. She had Meniere's disease and no psychiatric or neurological family history. Seven months before the visit, she started to claim not being informed of her husband's care plan from his care manager, which was suggestive of amnesia, as the care manager had informed her of the same. No other episodes suggested forgetfulness. Two months before the visit, she began to present delusions of theft, where her husband's visiting nurse stole things such as a passbook, a stamp, clothes, and dishes. Her delusion was not alleviated by changing the visiting nurse, and she finally called the police several times to claim the visiting nurse's unrealistic robbery. When referred to our department, she was admitted for diagnosis and treatment.

In the ward, her amnesia was apparent, as she often forgot new experiences. We did not confirm any apparent cognitive fluctuation, altered nighttime sleep behaviors, depressive mood, manic state, or hallucinations during hospitalization. She repeatedly claimed that the visiting nurse entered her house and stole her things. She also repeatedly showed irritability to medical staff because she forgot the reason for hospitalization. She finally claimed, “nurses in this ward steal my possessions” or “someone blows toxic gas to paralyze me.”

**Figure 2:**
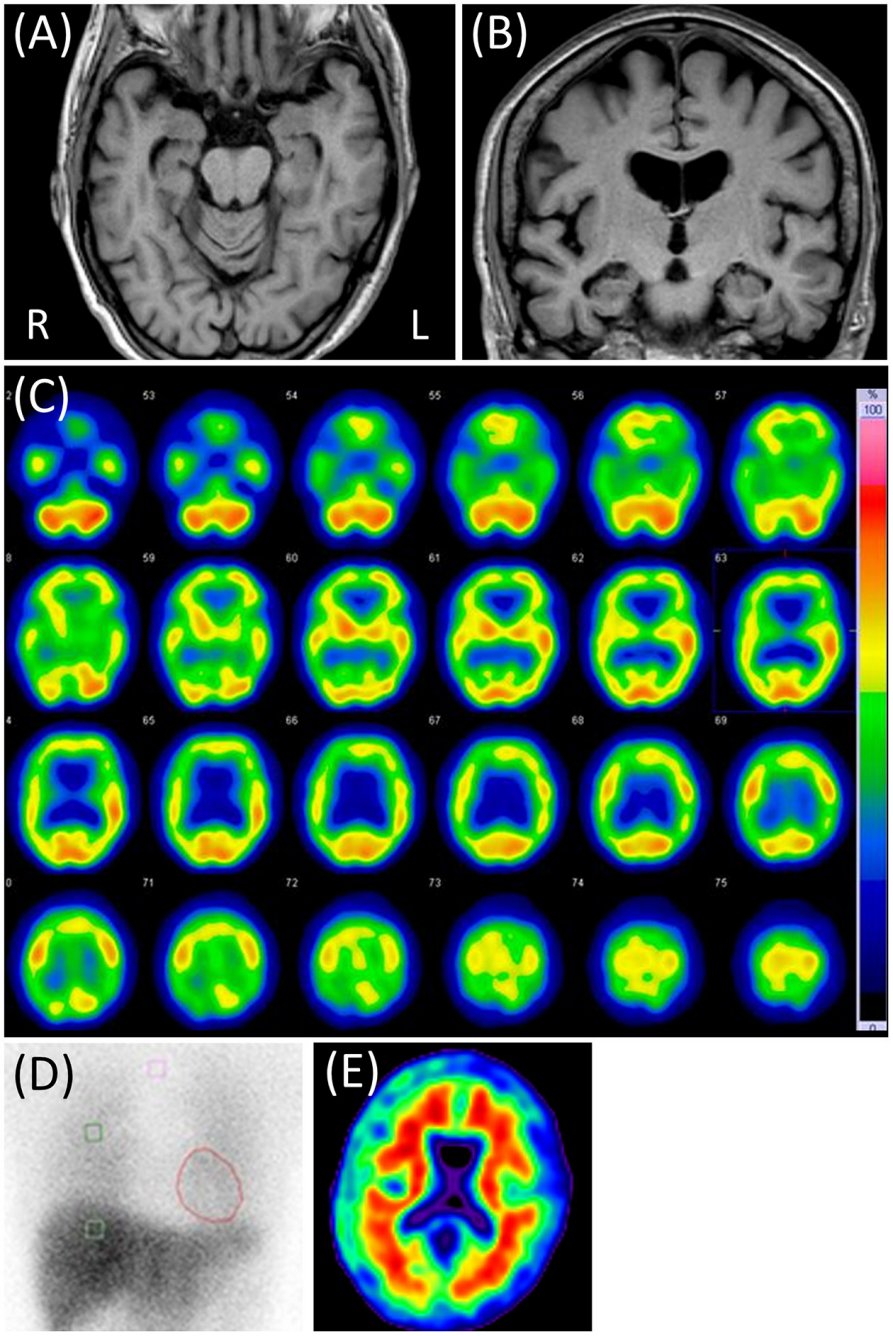
Case 2 images. **(A,B)** Brain magnetic resonance T1-weighted images showing slight diffuse brain atrophy. **(C)** N-isopropyl-p-[123I] iodoamphetamine single-photon emission computed tomography showing right-dominant hypoperfusion in the bilateral frontal, parietal, and temporal lobes. **(D)** Cardiac [123I]-metaiodobenzylguanidine scintigraphy. Heart-to-mediastinum ratios were 3.90 and 4.28 in early and delayed phases, respectively. The washout ratio was 26.0%. **(E)** Negative Florbetapir 18 positron emission tomography.

Neurological examination revealed her moderate hearing impairment, which often disturbs oral communication. It was mainly due to her Meniere's disease. Blood tests and electroencephalography did not reveal any abnormalities. In CSF, the cell count was 1/μL, and the protein concentration was 46 mg/dL. The MMSE score was 28 (two points lost in orientation), and the LM of the WMS-R score 9/0. The four subtests of the WAIS-III and the ADAS-J cog did not reveal any cognitive impairments other than memory impairment. The NPI-12 revealed dysphoria and anxiety apart from delusions. Brain MRI showed mild atrophy in her bilateral medial temporal and parietal lobes with slight periventricular white matter T2 hyperintensities and no cerebrovascular lesions. Right-dominant hypoperfusion in the frontal, parietal, and temporal lobes was observed on brain 123I-IMP SPECT, but cardiac 123I-MIBG scintigraphy results were normal (Figure 2).

Although we suspected some neurodegenerative disease, such as AD or AGD, because of the apparent amnesia and cerebral atrophy, we could not detect what neuropathology she had with the results above. On the contrary, we considered that her psychosis was too complicated to be attributed to neurodegeneration and diagnosed her with VLOSLP. Risperidone was prescribed to treat her delusions, but it induced headache and dizziness at 0.25 mg/day, so we switched to quetiapine up to 75 mg/day. After she complained of dry mouth, we decreased it to 37.5 mg/day. Soon thereafter, she started to complain of new persecutory delusions related to nurses in the ward. As hospitalization seemed to worsen her symptoms, we discharged her after putting in a visiting nurse to support her in taking prescribed drugs.

P-tau and t-tau levels in the CSF were 46 and 209 pg/mL, respectively. Florbetapir F 18 PET was also negative (Figure 2). APOE genotyping showed ε3/ε3. All findings were suggestive of the absence of AD pathology. Considering the above results and clinical presentation, she seemed to have some neurodegenerative pathology that was not AD. Her persecutory delusion toward the visiting nurse continued for ≥6 months after discharge. Thereafter, she was lost to follow-up.

The demographic characteristics and the results of principal tests (neuropsychological and others) in both cases are summarized in Table 1.

**Table 1:** Demographic characteristics, and results of neuropsychological and other tests in the two cases.

	**Case1**			**Case 2**
	**admission**	**1 year later**	**2 years later**	**admission**
Age	68	69	70	81
Gender	Female			Female
Education	high school			high school
MMSE				
Total score	28	25	23	28
Delayed Recall	3	0	0	3
ADAS-J.cog				
Total score	6.67	9.00	8.67	12.67
Word Recall	3.00	2.00	5.67	6.33
Word Recognition	2.67	4.00	1.00	2.33
False Recognition (number)	0	0	6	7
LM of WMS-R				
Immediate Recall	11	12	10	9
Delayed Recall	1	1	1	0
WAIS-III (index)				
Digit Symbol Substitution	12	15	NA	11
Block Design	8	9	NA	8
Digit Span	14	11	NA	11
Information	9	11	NA	11
CDR				
Total score	0.5	0.5	1	0.5
Sum of box	1.5	4	5	1.5
Neuropsychiatric Inventory 12				
Delusions	3	2	2	12
Hallucinations	0	0	0	0
Agitation/Aggression	0	0	0	0
Dysphoria/Depression	0	0	0	8
Anxiety	4	4	3	4
Euphoria/Elation	0	0	0	0
Apathy/Indifference	4	4	4	0
Disinhibition	0	0	0	2
Irritability/Lability	0	0	0	1
Aberrant Motor	0	0	0	0
Nighttime Behaviors	4	0	0	0
Appetite/Eating Disorders	8	8	4	0
Everyday Memory Check list				
Patient	8	8	8	12
Caregiver	9	9	22	10
Other tests				
CSF phosphorylated tau (pg/mL)	86	NA	NA	46
CSF total tau (pg/mL)	697	NA	NA	209
APOE genotyping	ε4/ε3	NA	NA	ε3/ε3

*MMSE, Mini-Mental State Examination; ADAS-J cog, Alzheimer's Disease Assessment Scale-Cognitive Subscale Japanese version; LM, Logical Memory; WMS-R, Wechsler Memory Scale-Revised; WAIS-III, Wechsler Adult Intelligence Scale Third Edition; CDR, Clinical Dementia Rating; CSF, cerebrospinal fluid; APOE, apolipoprotein E; NA, not applicable*.

## Discussion

Herein, we report two cases of clinically diagnosed VLOSLP. Both presented with persecutory delusions lasting >1 month with symptoms emerging after 60 years of age. Although their psychosis was prominent enough to disturb their functioning, the patients did not present any apparent affective symptoms. They scored 28 on the MMSE and maintained their activities of daily living. VLOSLP belongs to a group of non-affective and non-organic late-onset psychosis. As the original VLOSLP criteria did not mention clear checkpoints for the differential diagnosis between VLOSLP and psychosis secondary to organic diseases, most reports have employed clinician's decisions or the MMSE score to deny the presence of attributable organic brain disorders. The MMSE cut-off score varied around the mid-20's in different studies (19–21). Therefore, we diagnosed their psychoses as VLOSLP. They also met the criteria for delusional disorder using the Diagnostic and Statistical Manual of Mental Disorders Fifth Edition (DSM−5) (22). However, Case 1 showed abnormal AD biomarkers and progression to dementia. Therefore, we should consider methods to distinguish psychosis secondary to organic disorders, such as AD, from VLOSLP as primary psychosis.

Although both cases showed memory impairments in the detailed memory test, they preserved general cognition and independence in functional activities and managed their daily tasks by themselves. Their cognitive functions, other than memory, were generally preserved. They met the criteria for amnestic mild cognitive impairment (MCI) (23). Although amnestic MCI was originally considered to convert to AD dementia, its pathology is heterogeneous (24, 25); therefore, it should be assessed individually. The two cases presented different characteristics at several points. Case 1 showed a deficit only in LM, and Case 2 presented memory loss in daily living, impaired LM and ADAS-J cog. Case 1 did not show any particular imaging abnormalities, while Case 2 showed atrophy of the medial temporal lobes and hypoperfusion of the temporoparietal lobes. The traits of Case 2 were supportive of MCI due to AD (26), and at first we speculated that Case 2 rather than Case 1 had AD pathology. On the other hand, it was reported that amnestic MCI converting to AD dementia tends to lead to worse performance in delayed recall tasks with relatively preserved immediate recall tasks (27). Although Case 2 performed poorly even in the immediate recall task of the ADAS-J cog, Case 1 presented an apparent deficit only in the delayed LM recall. Thus, the performance difference in immediate and delayed memory tasks might support AD pathology in Case 1.

The delusions described were also puzzling with respect to pathology. Although both presented persecutory delusions, the themes of their delusions were different. Case 1 presented the delusion that someone intruded in her house and observed her. She also presented with a delusional mood related to prominent anxiety. Case 2 presented the extreme delusion that her husband's visiting nurse stole her things. Delusion of penetrating toxic gas was also observed during hospitalization. Delusions as behavioral and psychological symptoms of dementia frequently take the form of persecutory delusions (28). However, they typically revolve around predictable themes (29). Both cases had the idea that someone or something traversed barriers such as the walls of their houses or the ward. They were bizarre and typical of VLOSLP delusions (30). In particular, the delusion of observation in Case 1 and that related to toxic gas in Case 2 were far from the typical dementia-related delusions. In contrast, the delusion of theft in Case 2 is typical in patients with AD (31, 32). As for other comorbid neuropsychiatric symptoms, Case 1 presented apathy and Case 2 did not. Apathy without depression is a reliable predictor of progression to AD dementia in patients with amnestic MCI (33). However, this is not the case for patients with psychosis. Rather, the difference between the delusional themes of the two cases led us to assume that Case 2 had AD pathology.

AD biomarkers could clearly differentiate between the pathologies of the two cases. P-tau and t-tau in CSF were 86 and 697 pg/ml in Case 1 and 46 and 209 pg/ml in Case 2, respectively. Both were positive in Case 1 and negative in Case 2 for the diagnosis of AD. Amyloid PET was also positive in Case 1 and negative in Case 2. Therefore, we considered that Case 1 had AD pathology but not Case 2. Although we also speculated that Case 2 had other neurodegeneration due to brain atrophy and hypoperfusion, judging by our data, the type was unclear. There are few reports on the pathology of late-onset schizophrenia or VLOSLP. Several longitudinal studies on VLOSLP prognosis have clarified a tendency toward dementia (10–12). They support that VLOSLP contains prodromal neurodegenerative diseases. Pathological studies on late-onset schizophrenia and VLOSLP reported an association with AGD, LBD, and PART (8, 9). In Case 2, the amnesia emerging in the 80's, aggression, and hypoperfusion laterality might be suggestive of AGD (34, 35).

Neuropathological studies were pessimistic about the association between AD pathology and VLOSLP (8, 9). However, one report used an onset threshold of 45 years for the diagnosis of late-onset schizophrenia (8). Another report took delusion of theft, which was confirmed to be associated with AD, as an exclusion criterion for late-onset schizophrenia and delusional disorder (9). Therefore, there remains a possibility that some VLOSLP patients have prominent AD pathology, as Case 1 exhibited. Recent diagnostic criteria for AD put a value on CSF and amyloid PET to determine AD pathology and use the term “prodromal AD” for MCI with biomarker-confirmed AD pathology (36, 37). Case 1, where biomarkers indicated accumulated Aβ and tau in the brain, was also classified as prodromal AD confirmed by converting to AD dementia 2 years after the first visit. Thus, she might take the form of VLOSLP as a prodromal AD. It is essential to define whether early-stage psychosis results in or from MCI. Judging from her initial delusions, she claimed that the arrangement of things in her house was different from what she had thought at first. This seems to reflect her forgetting. These experiences might lead to the idea that anyone intruded on her house and moved things. As an explanation for delusion with dementia (38), her psychosis might be an attempt to fill in the gaps caused by her memory loss. Therefore, the psychosis in Case 1 would be secondary to MCI due to AD rather than the primary one with cognitive impairment. Fujishiro et al. also reported a case of autopsy-confirmed AD that showed delusional jealousy at an early stage (39). Therefore, severe delusions suggestive of primary psychosis can occur in the very early stages of AD. The two recent criteria of mild behavioral impairment (40) and psychosis in mild neurocognitive disorders (29), assuming cases with psychiatric symptoms in the early stage of AD, exclude severe psychosis such as primary psychotic disorder. Not to overlook AD, it may be necessary to consider the prodromal AD taking the form of VLOSLP, as the research criteria for prodromal DLB (41) mentioned listed late-onset psychosis as “psychiatric-onset” prodromal DLB.

Both cases were negative for LBD biomarkers. However, this does not mean that the patients had no LBD pathology. Cardiac MIBG scintigraphy and FP-CIT SPECT do not directly reflect α-synucleinopathy. There are not enough reports on their sensitivity for α-synucleinopathy in the prodromal LBD stage. Although the specific binding ratio was intact on FP-CIT SPECT in Case 1, the uptake in the left posterior putamen visually decreased, which might support a mixed pathology of AD and LBD. There are also many other neurodegenerative diseases such as AGD, PART, and limbic-predominant age-related TAR DNA-binding protein 43 encephalopathy (LATE), which can even coexist to form mixed neuropathologies. A longer follow-up period would be preferable to speculate neuropathology more correctly. In addition, there are already substantial reports about the correlation between amyloid PET, CSF biomarkers, and autopsy-confirmed pathologies (42–45). We might get closer to the pure VLOSLP by using biomarkers and paying careful attention to patient characteristics such as age and mixed pathologies (46).

In summary, we conclude with two key points: First, using only clinical assessments makes it challenging to speculate on the neuropathology of VLOSLP. Second, some patients with VLOSLP may have AD pathology. Prodromal AD may take the form of a VLOSLP. Further studies on VLOSLP with neuropathological biomarkers are needed.

## Data Availability Statement

The original contributions presented in the study are included in the article/supplementary material, further inquiries can be directed to the corresponding author/s.

## Ethics Statement

The studies involving human participants were reviewed and approved by Osaka University Clinical Research Review Committee. The patients/participants provided their written informed consent to participate in this study. Written informed consent was obtained from the individual(s) for the publication of any potentially identifiable images or data included in this article.

## Author Contributions

YS conducted neuropsychological assessments of the patients, collected the data, and wrote the initial draft of this article. MI conducted outpatient treatment for the patients. RN conducted the management of Case 2 during admission. KM and ES assessed all of the acquired nuclear images. HK, KY, MH, and MI participated in the discussion of the results and revised the manuscript. All authors discussed the diagnoses of both cases and approved the submitted manuscript.

## Funding

This work was supported by AMED under Grant Number JP21de0107001 and JSPS KAKENHI Grant Number T21K157300.

## Conflict of Interest

The authors declare that the research was conducted in the absence of any commercial or financial relationships that could be construed as a potential conflict of interest.

## Publisher's Note

All claims expressed in this article are solely those of the authors and do not necessarily represent those of their affiliated organizations, or those of the publisher, the editors and the reviewers. Any product that may be evaluated in this article, or claim that may be made by its manufacturer, is not guaranteed or endorsed by the publisher.

## References

[1] Bleuler M. Late schizophrenic clinical pictures. Fortschr Neurol Psychiatr. (1943) 15:259–90.

[2] Roth M Morrissey JD. Problems in the diagnosis and classification of mental disorder in old age; with a study of case material. J Ment Sci. (1952) 98:66–80. 10.1192/bjp.98.410.66 14898208

[3] Rabins P Pauker S Thomas J. Can schizophrenia begin after age 44? Compr Psychiatry. (1984) 25:290–3. 10.1016/0010-440X(84)90060-9 6734169

[4] Jeste DV Symonds LL Harris MJ Paulsen JS Palmer BW Heaton RK. Nondementia nonpraecox dementia praecox?: late-onset schizophrenia. Am J Geriatr Psychiatry. (1997) 5:302–17. 10.1097/00019442-199700540-00005 9363287

[5] Kay DW Roth M. Environmental and hereditary factors in the schizophrenias of age (‘late paraphrenia’) and their bearing on the general problem of causation in schizophrenia. J Ment Sci. (1961) 107:649–86. 10.1192/bjp.107.449.649 13752013

[6] Pearlson GD Kreger L Rabins PV Chase GA Cohen B Wirth JB. A chart review study of late-onset and early-onset schizophrenia. Am J Psychiatry. (1989) 146:1568–74. 10.1176/ajp.146.12.1568 2574011

[7] Howard R Rabins PV Seeman MV Jeste DV. Late-onset schizophrenia and very-late-onset schizophrenia-like psychosis: an international consensus. the international late-onset schizophrenia group. Am J Psychiatry. (2000) 157:172–8. 10.1176/appi.ajp.157.2.172 10671383

[8] Casanova MF Stevens JR Brown R Royston C Bruton C. Disentangling the pathology of schizophrenia and paraphrenia. Acta Neuropathol. (2002) 103:313–20. 10.1007/s00401-001-0468-6 11904750

[9] Nagao S Yokota O Ikeda C Takeda N Ishizu H Kuroda S. Argyrophilic grain disease as a neurodegenerative substrate in late-onset schizophrenia and delusional disorders. Eur Arch Psychiatry Clin Neurosci. (2014) 264:317–31. 10.1007/s00406-013-0472-6 24272318

[10] Brodaty H Sachdev P Koschera A Monk D Cullen B. Long-term outcome of late-onset schizophrenia: 5-year follow-up study. Br J Psychiatry. (2003) 183:213–9. 10.1192/bjp.183.3.213 12948993

[11] Kørner A Lopez AG Lauritzen L Andersen PK Kessing LV. Late and very-late first-contact schizophrenia and the risk of dementia-a nationwide register based study. Int J Geriatr Psychiatry. (2009) 24:61–7. 10.1002/gps.2075 18561206

[12] Stafford J Dykxhoorn J Sommerlad A Dalman C Kirkbride JB Howard R. Association between risk of dementia and very late-onset schizophrenia-like psychosis: a Swedish population-based cohort study. Psychol Med. (2021) 25:1–9. 10.1017/S0033291721002099. [Epub ahead of print]. 34030750 PMC9975996

[13] Palmqvist S Zetterberg H Mattsson N Johansson P. Alzheimer's Disease Neuroimaging Initiative Minthon L. Detailed comparison of amyloid PET and CSF biomarkers for identifying early Alzheimer disease. Neurology. (2015) 85:1240–9. 10.1212/WNL.0000000000001991 26354982 PMC4607601

[14] Thomas AJ Donaghy P Roberts G Colloby SJ Barnett NA Petrides G. Diagnostic accuracy of dopaminergic imaging in prodromal dementia with Lewy bodies. Psychol Med. (2019) 49:396–402. 10.1017/S0033291718000995 29692275 PMC6331684

[15] Fujishiro H Ota K Yamagata M Ito T Hieda S Suga H. Early diagnosis of prodromal dementia with Lewy bodies using clinical history of probable REM sleep behavior disorder and cardiac 123I-MIBG scintigraphy in memory clinics. Psychogeriatrics. (2021) 21:288–95. 10.1111/psyg.12662 33565213

[16] Van Everbroeck B Green AJE Vanmechelen E Vanderstichele H Pals P Sanchez-Valle R. Phosphorylated tau in cerebrospinal fluid as a marker for Creutzfeldt–Jakob disease. J Neurol Neurosurg Psychiatry. (2002) 73:79–81. 10.1136/jnnp.73.1.79 12082054 PMC1757299

[17] Nishimura T Takeda M Nakamura Y Yosbida Y Arai H Sasaki H. Basic and clinical studies on the measurement of Tau protein in cerebrospinal fluid as a biological marker for alzheimer's disease and related disorders: multicenter study in Japan. Methods Find Exp Clin Pharmacol. (1998) 20:227–35. 9646285

[18] Humpel C. Identifying and validating biomarkers for alzheimer's disease. Trends Biotechnol. (2011) 29:26–32. 10.1016/j.tibtech.2010.09.007 20971518 PMC3016495

[19] Psarros C Theleritis CG Paparrigopoulós TJ Politis AM Papadimitriou GN. Amisulpride for the treatment of very-late-onset schizophrenia-like psychosis. Int J Geriatr Psychiatry. (2009) 24:518–22. 10.1002/gps.2146 19072747

[20] Lam CCSF Reeves SJ Stewart R Howard R. Service and treatment engagement of people with very late-onset schizophrenia-like psychosis. BJPsych Bull. (2016) 40:185–6. 10.1192/pb.bp.115.051599 27512585 PMC4967775

[21] Howard R Cort E Bradley R Harper E Kelly L Bentham P. Antipsychotic treatment of very late-onset schizophrenia-like psychosis (ATLAS): a randomised, controlled, double-blind trial. Lancet Psychiatry. (2018) 5:553–63. 10.1016/S2215-0366(18)30141-X 29880238 PMC6015223

[22] American Psychiatric Association. Diagnostic And Statistical Manual Of Mental Disorders. 5^th^ ed. Arlington: American Psychiatric Publishing, Inc. (2013). 10.1176/appi.books.9780890425596

[23] Winblad B Palmer K Kivipelto M Jelic V Fratiglioni L Wahlund LO. Mild cognitive impairment-beyond controversies, towards a consensus: report of the international working group on mild cognitive impairment. J Intern Med. (2004) 256:240–6. 10.1111/j.1365-2796.2004.01380.x 15324367

[24] Schneider JA Arvanitakis Z Leurgans SE Bennett DA. The neuropathology of probable alzheimer disease and mild cognitive impairment. Ann Neurol. (2009) 66:200–8. 10.1002/ana.21706 19743450 PMC2812870

[25] Dugger BN Davis K Malek-Ahmadi M Hentz JG Sandhu S Beach TG. Neuropathological comparisons of amnestic and nonamnestic mild cognitive impairment. BMC Neurol. (2015) 15:146. 10.1186/s12883-015-0403-4 26289075 PMC4545878

[26] Albert MS DeKosky ST Dickson D Dubois B Feldman HH Fox NC. The diagnosis of mild cognitive impairment due to alzheimer's disease: recommendations from the national institute on aging-alzheimer's association workgroups on diagnostic guidelines for alzheimer's disease. Alzheimers Dement. (2011) 7:270–9. 10.1016/j.jalz.2011.03.008 21514249 PMC3312027

[27] De Simone MS Perri R Fadda L De Tollis M Turchetta CS Caltagirone C. Different deficit patterns on word lists and short stories predict conversion to alzheimer's disease in patients with amnestic mild cognitive impairment. J Neurol. (2017) 264:2258–67. 10.1007/s00415-017-8623-8 28948357

[28] Cipriani G Danti S Vedovello M Nuti A Lucetti C. Understanding delusion in dementia: a review. Geriatr Gerontol Int. (2014) 14:32–9. 10.1111/ggi.12105 23879399

[29] Cummings J Pinto LC Cruz M Fischer CE Gerritsen DL Grossberg GT. Criteria for psychosis in major and mild neurocognitive disorders: international psychogeriatric association (IPA) consensus clinical and research definition. Am J Geriatr Psychiatry. (2020) 28:1256–69. 10.1016/j.jagp.2020.09.002 32958332 PMC7669601

[30] Howard R Castle D O'Brien J Almeida O Levy R. Permeable walls, floors, ceilings and doors. partition delusions in late paraphrenia. Int J Geriatr Psychiatry. (1992) 7:719–24. 10.1002/gps.930071006

[31] Ikeda M Shigenobu K Fukuhara R Hokoishi K Nebu A Maki N. Delusions of Japanese patients with alzheimer's disease. Int J Geriatr Psychiatry. (2003) 18:527–32. 10.1002/gps.864 12789674

[32] Seeman MV. Understanding the delusion of theft. Psychiatr Q. (2018) 89:881–9. 10.1007/s11126-018-9588-1 29956101

[33] Palmer K Di Iulio F Varsi AE Gianni W Sancesario G Caltagirone C. Neuropsychiatric predictors of progression from amnestic-mild cognitive impairment to alzheimer's disease: the role of depression and apathy. J Alzheimers Dis. (2010) 20:175–83. 10.3233/JAD-2010-1352 20164594

[34] Togo T Isojima D Akatsu H Suzuki K Uchikado H Katsuse O. Clinical features of argyrophilic grain disease: a retrospective survey of cases with neuropsychiatric symptoms. Am J Geriatr Psychiatry. (2005) 13:1083–91. 10.1097/00019442-200512000-00008 16319301

[35] Adachi T Saito Y Hatsuta H Funabe S Tokumaru AM Ishii K. Neuropathological asymmetry in argyrophilic grain disease. J Neuropathol Exp Neurol. (2010) 69:737–44. 10.1097/NEN.0b013e3181e5ae5c 20535032

[36] Dubois B Feldman HH Jacova C Hampel H Molinuevo JL Blennow K. Advancing research diagnostic criteria for alzheimer's disease: the IWG-2 criteria. Lancet Neurol. (2014) 13:614–29. 10.1016/S1474-4422(14)70090-0 24849862

[37] Jack CR Bennett DA Blennow K Carrillo MC Dunn B Haeberlein SB. NIA-AA research framework: toward a biological definition of alzheimer's disease. Alzheimers Dement. (2018) 14:535–62. 10.1016/j.jalz.2018.02.018 29653606 PMC5958625

[38] Cohen-Mansfield J Golander H Cohen R. Rethinking psychosis in dementia: an analysis of antecedents and explanations. Am J Alzheimers Dis Other Demen. (2017) 32:265–71. 10.1177/1533317517703478 28468553 PMC10852834

[39] Fujishiro H Iritani S Hattori M Sekiguchi H Matsunaga S Habuchi C. Autopsy-confirmed hippocampal-sparing alzheimer's disease with delusional jealousy as initial manifestation. Psychogeriatrics. (2015) 15:198–203. 10.1111/psyg.12105 25737011

[40] Ismail Z Smith EE Geda Y Sultzer D Brodaty H Smith G. Neuropsychiatric symptoms as early manifestations of emergent dementia: provisional diagnostic criteria for mild behavioral impairment. Alzheimers Dement. (2016) 12:195–202. 10.1016/j.jalz.2015.05.017 26096665 PMC4684483

[41] McKeith IG Ferman TJ Thomas AJ Blanc F Boeve BF Fujishiro H. Research criteria for the diagnosis of prodromal dementia with lewy bodies. Neurology. (2020) 94:743–55. 10.1212/WNL.0000000000009323 32241955 PMC7274845

[42] Tapiola T Alafuzoff I Herukka SK Parkkinen L Hartikainen P Soininen H. Cerebrospinal fluid β-amyloid 42 and tau proteins as biomarkers of alzheimer-type pathologic changes in the brain. Arch Neurol. (2009) 66:382–9. 10.1001/archneurol.2008.596 19273758

[43] Mattsson-Carlgren N Andersson E Janelidze S Ossenkoppele R Insel P Strandberg O. Aβ deposition is associated with increases in soluble and phosphorylated tau that precede a positive Tau PET in alzheimer's disease. Sci Adv. (2020) 6:eaaz2387. 10.1126/sciadv.aaz2387 32426454 PMC7159908

[44] Murray ME Lowe VJ Graff-Radford NR Liesinger AM Cannon A Przybelski SA. Clinicopathologic and 11C-pittsburgh compound B implications of thal amyloid phase across the alzheimer's disease spectrum. Brain. (2015) 138:1370–81. 10.1093/brain/awv050 25805643 PMC4407190

[45] Clark CM Schneider JA Bedell BJ Beach TG Bilker WB Mintun MA. Use of florbetapir-PET for imaging β-amyloid pathology. JAMA. (2011) 305:275–83. 10.1001/jama.2010.2008 21245183 PMC7041965

[46] Ossenkoppele R Jansen WJ Rabinovici GD Knol DL van der Flier WM van Berckel BNM. Prevalence of amyloid PET positivity in dementia syndromes: a meta-analysis. JAMA. (2015) 313:1939–49. 10.1001/jama.2015.4669 25988463 PMC4517678

